# Sex-specific relationships between face memory and the N170 component in event-related potentials

**DOI:** 10.1093/scan/nsaa059

**Published:** 2020-05-05

**Authors:** Hadiseh Nowparast Rostami, Andrea Hildebrandt, Werner Sommer

**Affiliations:** 1 Institut für Psychologie, Humboldt-Universität zu Berlin, 12489 Berlin, Germany; 2 Department of Physics, Centre for Nonlinear Studies, Institute of Computational and Theoretical Studies, Hong Kong Baptist University, Kowloon Tong, Hong Kong; 3 Department of Psychology, Carl von Ossietzky Universtät Oldenburg, 26129 Oldenburg, Germany

**Keywords:** face memory, N170, sex differences, individual differences

## Abstract

At the group level, women consistently perform better in face memory tasks than men and also show earlier and larger N170 components of event-related brain potentials (ERP), considered to indicate perceptual structural encoding of faces. Here we investigated sex differences in the relationship between the N170 and face memory performance in 152 men and 141 women at group mean and individual differences levels. ERPs and performance were measured in separate tasks, avoiding statistical dependency between the two. We confirmed previous findings about superior face memory in women and a—sex-independent—negative relationship between N170 latency and face memory. However, whereas in men, better face memory was related to larger N170 components, face memory in women was unrelated with the amplitude or latency of the N170. These data provide solid evidence that individual differences in face memory within men are at least partially related to more intense structural face encoding.

## Introduction

### Sex differences in face memory

Female superiority in face processing is a well-established finding. This holds true for recognizing both emotional facial expressions (McClure, 2000) and facial identities (Herlitz and Lovén, 2013; Heisz *et al.*, 2013). Sex differences in face memory seem to be present already in childhood (Gur *et al.*, 2012; Fuhrmann *et al.*, 2016). Based on a sample of about 800 adults who completed an extensive test battery, Sommer *et al.* (2013) reported women to outperform men in both face perception and face memory; the effect size of female superiority in face memory increased across adult age and was considerable (around 1 s.d.) even after accounting for individual differences in general cognitive functioning. The suggestion that female superiority holds true especially for own sex faces (Herlitz and Lovén, 2013) is not consistently supported (Hoheisel and Kryspin-Exner, 2005; Sommer *et al.*, 2013). Therefore, the current consensus seems to be that there is female superiority in the abilities to perceive, learn and recognize faces of both sexes, which is independent of general cognitive functioning.

### The N170 component, its significance for face recognition and sex differences

Although sex differences in face memory are well established, little is known about their neural correlates. A large number of event-related brain potential (ERP) studies have shown that the N170 component in the ERP waveform is usually larger in response to faces than to non-face objects (e.g. Bentin *et al.*, 1996; Eimer, 2011; Ganis *et al.*, 2012) and, therefore, may be face-specific. The N170 is a negative-going ERP deflection at the inferior occipitotemporal scalp, peaking around 170 ms after stimulus onset. In line with the functional model of face recognition proposed by Bruce and Young (1986), the N170 component has been suggested to reflect the perceptual encoding of facial structures (e.g. Bentin *et al.*, 1996; Eimer, 2000), for example, because it often increases in amplitude and is delayed in peak latency when structural encoding is challenged by presenting the face stimuli upside down. The N170 seems to be generated within the neuronal core system of face recognition (Haxby and Gobbini, 2011), that is, in the fusiform (e.g. Deffke *et al.*, 2007), occipitotemporal or superior temporal gyri (e.g. Nguyen and Cunnington, 2014). Moreover, several studies on the relation between individual face cognition abilities and ERPs consistently revealed a negative correlation of the ability to accurately perceive and remember faces with the N170 latency, that is, with the time demands for structural face encoding (Herzmann *et al.*, 2010; Kaltwasser *et al.*, 2014; Nowparast Rostami *et al.*, 2017).

Therefore, it seems reasonable to ask whether the well-established sex differences in face memory can be—at least partially—explained by sex differences in the structural encoding of faces not only on the group level but on the level of individual differences. Hitherto, only a few studies have considered sex differences in the N170 and addressed sex-specific relationships between the N170 and face memory using different approaches. Based on data from 28 participants, Sun *et al.* (2010) reported that early encoding of face structure is modulated by task demands to a larger degree in women than in men; females showed larger N170 amplitudes when discriminating female face gender in upright *vs* upside-down orientation as compared to male faces, but there was no such task effect in men. Therefore, it seems that men and women use different strategies already during early stages of face processing. In an oddball task, Choi *et al.* (2015) reported larger N170 amplitudes for women (*N* = 10) in response to emotional target faces than to neutral non-target faces, whereas in men (*N* = 12) the N170 amplitudes did not differ between targets and non-targets. Recently, Sun *et al.* (2017) investigated sex differences in behavioral and neural responses to face stimuli and their relationship (*N* = 32). Using a modified delayed matching-to-sample task, the authors studied the neural correlates of female superiority in face recognition using ERPs. Overall, women were faster and more accurate than men in recognizing target faces and showed shorter peak latencies of the P100 and N170 components than men, as well as larger amplitudes of the late positive P300 component. In addition, only in women reaction times (RTs) to faces were positively correlated with N170 latencies. Focusing on the own-gender bias, Wolff *et al.* (2014) reported larger N170 amplitudes in female compared with male participants. Furthermore, they observed a face-gender effect for male participants, reflecting larger N170 amplitude for female *vs* male faces, during their learning phase. The authors also examined the neural correlates of the own-gender bias in face memory. However, analysis of the N170 did not reveal strong evidence for different processing of own- and other-gender faces.

Together, these findings indicate that the N170 is related to face memory performance and this relationship may be sex-specific. Unfortunately, the previous studies suffer from the shortcoming that the number of participants was much too small to allow firm conclusions about individual differences in females *vs* males; according to our own previous studies, correlations between N170 and performance are small and hence require considerable power and sample size. In addition, these previous studies used ERP and performance data from the same trials, which makes them statistically dependent and therefore vulnerable to artifacts, such as momentary lapses in attention or erroneous responses.

### Aims of the present study

The evidence summarized above suggests that women exhibit shorter latencies and larger amplitudes in the N170 component; therefore, one may assume that faster and more intense analysis of facial structures may be a neurocognitive mechanism contributing to female superiority in face memory. In the present study, we tested this hypothesis in a large sample of participants who completed multiple tasks of face memory that were independent from the tasks used for ERP recording. In contrast to previous studies, we used a latent variable approach and structural equation modeling (SEM) which have the advantage of accounting for measurement error and the specificity of measurement methods. Specifically, we addressed the following research questions. Aiming to replicate previous findings, we investigated female superiority in face memory performance, parameterized as a latent variable. We modeled also N170 latency and amplitude as latent variables and tested whether women have shorter latencies and larger amplitudes, hence faster and possibly more intense structural encoding of faces than men. Addressing the main question of the present study, we regressed face memory onto N170 latency and amplitude for men and women simultaneously in multiple group SEM and investigated the relationships between the N170 parameters and face memory, testing whether these relationships are different for females and males.

## Methods

### Overview

In order to investigate our research questions, we merged two published datasets with a large overlap in face memory tasks and EEG recordings. Primary reports on Datasets 1 (*N* = 198) and 2 (*N* = 95) have been published by Nowparast Rostami *et al.* (2017) and Kaltwasser *et al.* (2014), respectively. The datasets were obtained to address related but different research questions about brain behavior relationships. However, neither of the previous reports had studied sex differences. Both previous studies had been conducted in accord with the Declaration of Helsinki and had been approved by the ethics committee of the Instituit für Psychologie of the Humboldt-Universität zu Berlin.

### Participants

The total sample consisted of *N* = 293 healthy Caucasian young adults (aged 18–40 years) with heterogeneous occupational and educational backgrounds, including 152 men (mean age 27.30 ± 5.29 years; 87.50% right-handed) and 141 women (mean age 27.45 ± 5.16; 86.52% right-handed). All participants had normal or corrected-to-normal visual acuity.

### Stimuli

The same set of stimuli had been used in obtaining both datasets consisting in black and white portraits (50% women) from frontal views with direct gaze, neutral facial expressions and without distinctive features like glasses or tattoos. All photographs were taken from two external databases (Endl *et al.*, 1998; Lundqvist *et al.*, 1998) and our own database (Hildebrandt *et al.*, 2010) and were initially unfamiliar to the participants. All portraits were fitted into a vertical ellipse of 259 × 388 pixels (7.0 × 10.2 cm) and matched in luminance.

### Procedure

Data acquisition for both datasets consisted of two parts. Participants first completed a battery of psychometric tasks, followed after about 1 week by a learning-recognition experiment with faces, including EEG recordings. Here, we only report data from the tasks of interest for the present research questions.

#### Psychometric study

Aiming to capture the accuracy of face memory, the test battery in both datasets included three different tasks, which had been psychometrically tested and validated (Hildebrandt *et al.*, 2010, 2011, 2012, 2013; Wilhelm *et al.*, 2010). Below, we provide a brief description of the tasks and refer to previous publications for more details.

##### Learning and recognition of faces

First, 30 faces were presented in 2 arrays of 15 simultaneously displayed stimuli, and participants were instructed to memorize them. Then, recognition of the learned faces was tested after an intervening task by presenting each learned face next to a distractor. Participants should identify the learned face by pressing a spatially corresponding left or right button, depending on the position of the familiar face.

##### Decay rate of learned faces

Approximately 90 min after the previous learning and recognition task, participants were again shown each face that had been learned in the previous task next to an unfamiliar face. Participants should indicate the learned face by pressing the spatially corresponding button.

##### Incidental face memory

In 46 trials an unfamiliar face was presented together with a face that had been shown in a previous face perception task (not relevant for the present work) without explicit memorization instruction. Participants were to indicate the previously seen—incidentally learned—faces by button presses.

#### E‌EG study

The EEG data in both datasets were recorded during a separate face recognition task in a prime-target paradigm; all familiar faces for these tasks had been explicitly memorized prior to the recognition phase. There was no overlap between the faces used in the psychometric and EEG parts of the study. In Datasets 1 and 2, participants had to memorize 12 or 10 faces, each being presented for 5 or 45 s, respectively. Participants learned the faces by writing or naming some properties describing the stimulus face, for example, ‘big nose’, ‘thin lips’, etc. Then, each of these faces was presented together with an unfamiliar distractor, while the learned face was to be indicated by a button press. This block was repeated with a different ordering of learned faces and new unfamiliar faces until accuracy was at 100%.

After the learning phase, the face recognition task followed. Participants performed a face familiarity decision, with choice responses to the second of two faces. The first face was the same or different from the second face, yielding a repetition priming paradigm with the conditions primed and unprimed familiar faces (PF, UF) and primed and unprimed unfamiliar faces (PUF, UPUF). Since the priming manipulation is not relevant here and also did not affect the data of concern, no further details (to be found, in Kaltwasser *et al.*, 2014 and Nowparast Rostami *et al.*, 2017) will be provided.

**Fig. 1:**
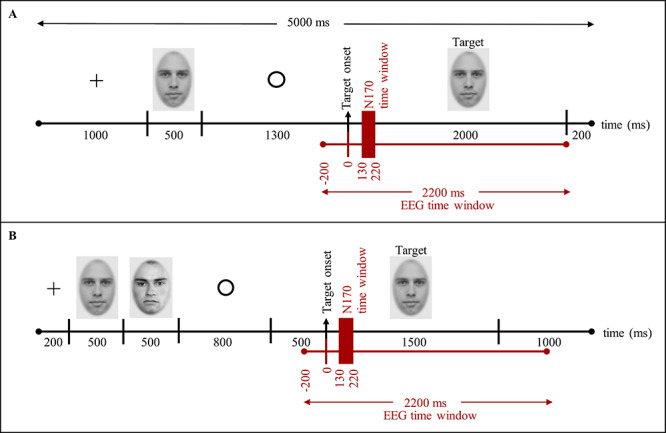
Trial schemes of the face recognition tasks applied for collecting the EEG Dataset 1 (A) and Dataset 2 (B).

Per condition, there were 72 and 80 trials in Datasets 1 and 2, respectively. In the experiment yielding Dataset 1, each trial began with a black fixation cross presented for 1 s, followed by a prime face for 500 ms. The prime was then replaced by a fixation circle for 1.3 s, followed by the target stimulus for 2 s and an inter-trial interval of 200 ms (see Figure 1A). In the experiment yielding Dataset 2, each trial began with a black fixation cross for 200 ms, followed by a prime face for 500 ms. A new unfamiliar face (mask) was shown for 500 ms and then replaced by a fixation circle for 800 ms, followed by a blank screen for 500 ms. The target stimulus was shown for 1500 ms; the inter-trial interval was 1 s (see Figure 1B). In both experiments participants had to respond to the target face as quickly and accurately as possible.

### Psychophysiological recordings

EEG was recorded from 40 Ag/AgCl electrodes mounted in an elastic cap (Easy cap, Brain Products, Germany), including AFz electrode as ground. Two electrodes were positioned directly on the left (reference) and right mastoid. In order to monitor blinks and eye movements, electrooculograms were recorded from electrodes positioned at the outer canthi of both eyes and a right infraorbital electrode. Impedances of all electrodes were kept below 5 kΩ. EEG was amplified using BrainAmp DC amplifiers (Brain Products, Germany) with 0.1 μV resolution, 5000 Hz sampling rate, 0.1 Hz low cutoff and 1000 Hz high cutoff. Data were recorded in Brain Vision Recorder (Brain Products, Germany), down-sampled to 1000 Hz.

### Data treatment

#### Psychometric data

The indicators of performance accuracy in each task were the proportions of correct responses. Outliers were identified by screening accuracy in each task in uni- and bivariate distributions for observations located outside the whiskers of the boxplots (*g* = 1.5; Tukey, 1977) and set to missing. All missing values were replaced using multiple random imputations (e.g. Allison, 2001), implemented in the R package mice by Buuren and Groothuis-Oudshoorn (2011). Because our aim was to test sex differences, we inspected univariate distributions in group comparison and observed no differences in variance and distribution shapes across the groups.

#### Psychophysiological data

EEG data were pre-processed using the EEGLAB toolbox (Delorme and Makeig, 2004). In the first step, blinks and eye movements were removed by means of independent component analysis (function: runica(); algorithm: Infomax (Gradient)). As a guideline for selecting and rejecting the artifact components, we used SASICA (EEGLAB plugin; Chaumon *et al.*, 2015). Then, data were filtered by a low-pass Hamming windowed sinc FIR filter with 40 Hz cutoff and 12 dB/oct roll-offs and recalculated to average reference.

After segmenting the continuous EEG into 5 s epochs, starting from the onset of the first fixation stimulus, the linear trends were removed from each epoch. Because in the prime-target paradigm the first face stimulus (prime) is task-irrelevant, we focused on the ERP responses to target stimuli, selecting the epochs from 200 ms pre- to 2000 ms post-target onset; all epochs were referred to a 200 ms pre-target baseline. Epochs with amplitude differences >120 μV or amplitudes exceeding ±80 μV were considered artifacts and excluded. In addition, trials with missing or incorrect responses, RTs < 200 msec, or outlier RTs, detected by Tukey’s outlier filter (see above), were discarded.

### Psychometric modeling

We conducted single- (e.g. Bollen and Long, 1993) and multigroup mean and covariance structure analysis (e.g. Little *et al.*, 2007) by using the lavaan package (Rosseel, 2012) in the R software for statistical computing (R Core Team, 2014). Model quality was assessed by multiple statistical tests and fit indices: χ^2^ statistics, the root mean square error of approximation (RMSEA < 0.08), standardized root mean square residual (SRMR < 0.08) and the comparative fit index (CFI > 0.95). For model comparisons we relied on χ^2^ difference tests. Latent variables were standardized for identification purpose.

## Results

### Sex difference in face memory accuracy

We modeled face memory as a latent variable and confirmed its previously established measurement model (Hildebrandt *et al.*, 2010, 2011, 2012, 2013; Wilhelm *et al.*, 2010). In this model, performance indicators are the proportion of correct responses across trials within each face memory task, as described above. In order to test whether the sex difference in face memory ability is replicable, the standardized latent face memory factor was regressed onto the dummy-coded variable sex (coding: male = 0, female = 1). The model showed an excellent fit: χ^2^ [2] = 0.08, *P* = 0.95, CFI = 1, RMSEA = 0.000 and SRMR = 0.003. Factor loadings ranged between 0.50 and 0.87 and were significant (see Figure 3A). In this model, there was a significant relationship between sex and face memory (β = 0.31, *P* = 0.01). Given the standardized latent variable and the dummy coding of sex, the β coefficient indicates that women performed almost one-third of a standard deviation better than men.

### Sex difference in the N170 latency and amplitude

**Fig. 2:**
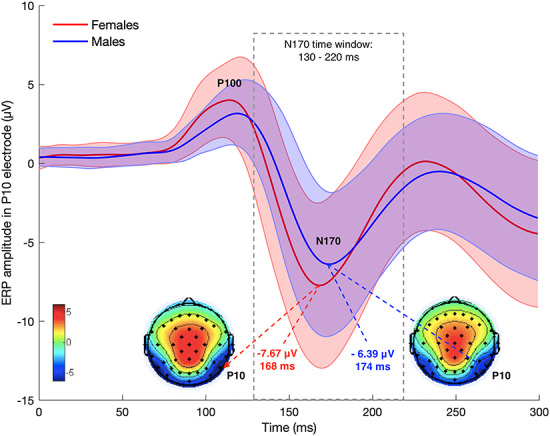
Amplitudes and topographies of the N170 component for females and males. Colors in topographies are calibrated in μV.

Figure 2 depicts amplitudes and latencies of the N170 component for women and men. The N170 for recognized faces in each condition (PF, UPF, PUF, UPUF) was measured as peak latency and peak amplitude between 130 and 220 ms after target onset at the P10 electrode. Before combining the two datasets, we conducted several *t*-tests to compare the average N170 latency and amplitude in each condition between the two datasets. The *t*-tests indicated the N170 latencies to be significantly longer in Dataset 1 than in Dataset 2 in all conditions (174.31 *vs* 162.66 ms in PF; 176.19 *vs* 163.42 ms in UPF; 174.45 *vs* 163.87 ms in PUF; 176.79 *vs* 164.02 ms in UPUF). Moreover, N170 amplitudes were significantly larger in Dataset 1 than Dataset 2 (−9.30 *vs* −7.18 μV in PF; −9.21 *vs* −6.66 μV in UPF; −8.92 *vs* −6.63 μV in PUF; −9.46 *vs* −7.08 μV in UPUF). As outlined in the task descriptions of the EEG studies, the trial designs were somewhat different in the two experiments, which may have resulted in some (additional) adaptation effects (e.g. Kovacs *et al.*, 2006) in Dataset 2. In order to adjust for these differences, Dataset 2 was transformed by adding the average difference values measured at the sample level to the observed values of each participant. Because adding a constant value to all observations of a variable does not lead to changes in rank order between individuals, this data transformation did not affect our results but helped to achieve comparability of means across the two datasets. Please note that another approach to unifying the datasets would be a centering of all variables on their study-specific means. The centering approach leads to the same inferential conclusions as the one selected here.

**Fig. 3:**
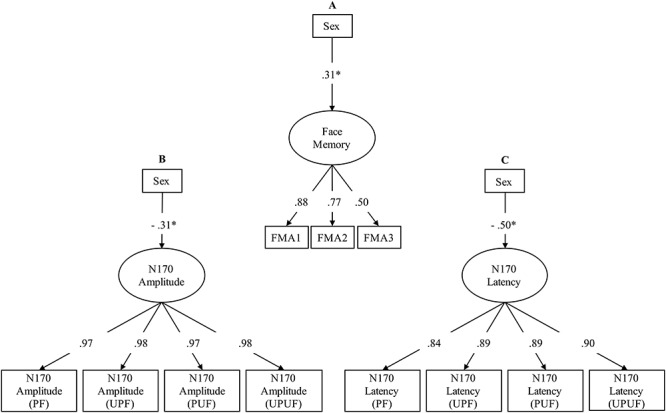
Model of testing sex differences in (A) face memory accuracy, (B) N170 amplitude and (C) N170 latency. ^*^Significant relationships (*P* < 0.05).

**Fig. 4:**
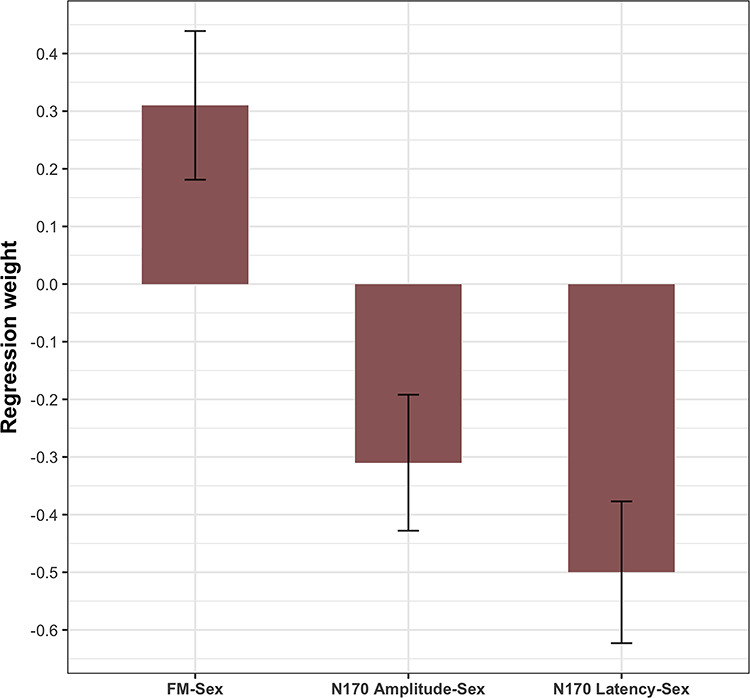
Summary of sex differences on latent factor means for neural and behavioral measures in standard deviation units.

To test sex differences in the average N170 (N170 latency: 168 ms in female *vs* 174 ms in males; N170 amplitude: −7.67 μV in female *vs* −6.39 μV in males), we estimated latent variables of N170 latency and amplitude in separate models. To this aim we used four indicators, namely, the N170 latency and amplitude measures in the four experimental conditions described above. The standardized latent factors were then regressed onto the dummy-coded manifest variable sex (male = 0, female = 1). Both models showed excellent fits. For the N170 latency, the fit was χ^2^ [5] = 1.54, *P* = 0.91, CFI = 1, RMSEA = 0.000 and SRMR = 0.007 and for amplitude, χ^2^ [5] = 7.88, *P* = 0.16, CFI = 0.999, RMSEA = 0.044 and SRMR = 0.004. Factor loadings ranged between 0.84 and 0.90 in the N170 latency model and between 0.97 and 0.98 in the N170 amplitude model (see Figure 3). There were significant relationships of sex with N170 latency (β = −0.50, *P* < 0.01) and with N170 amplitude (β = −0.31, *P* = 0.01). Given standardized latent variables and the dummy coding, these results indicate that the N170 occurs half of a standard deviation earlier and is one-third of a standard deviation larger (more negative) in females as compared with males. Figure 4 provides a summary of sex effects on the latent N170 and face memory factors.

### Sex differences in the association between face memory and the N170

#### Relationship of face memory and N170 latency

Next, we applied structural equation modeling to test the sex-independent contribution of the N170 latency to individual differences in face memory accuracy. The brain–behavior relationship was tested as regression path between the latent factors, established in the measurement models described above. The model showed very good fit to the data: χ^2^ [13] = 7.30, *P* = 0.89, CFI = 1, RMSEA = 0.000 and SRMR = 0.023. The brain–behavior relation revealed that shorter N170 latencies were associated with better face memory performance (β = −0.22, *P* < 0.01).

**Fig. 5:**
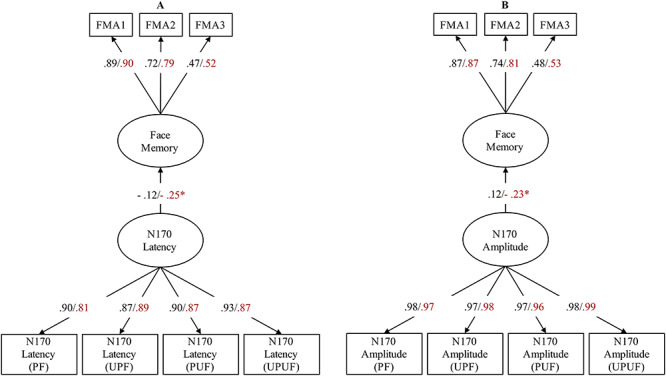
Multiple group models of testing brain–behavior relations in males and females. Face memory is regressed onto N170 latency in model (A) and N170 amplitude in model (B). The factor loadings and regression weights are shown for both males and females. The left and right numbers belong to the female and male groups, respectively (female/male).

Prior to investigating sex differences in the association between face memory and N170 latency, we tested the measurement invariance within the structural model depicted in Figure 5 across sex groups. This is a necessary precondition for group comparisons at the level of latent factors (Little *et al.*, 2007) in order to ascertain equivalent meaning of latent factors in the compared groups. Measurement invariance was tested by constraining the model parameters to equality across groups in a stepwise manner. First, we tested whether the patterns of factor loadings were equivalent for both sexes. The model with invariant configuration showed excellent fit (see Table 1, Line 1). Second, we fixed all corresponding factor loadings in the model to equality across groups. This metric invariant model also fitted the data very well and not significantly worse than the configural invariant model (see Table 1, Line 2). Hence the tasks measured the latent variables with similar discriminative power in both men and women. Third, also indicator intercepts were constrained to equality across groups. This scale invariant model revealed very good fit that did not significantly differ from the metric invariant model (see Table 1, Line 3), suggesting that also task difficulty is comparable across sexes. Taken together, the model testing measurement invariance showed that latent factor means and brain–behavior relations can be tested across sex groups because the tasks have equivalent meaning in the two groups. That is, group comparisons of regression weights will not be biased by different measurement characteristics across groups.

**Table 1:** Results of invariance testing.

Models		χ^2^	*df*	CFI	RMSEA	SRMR	Δχ^2^ (Δ*df*)	*P*
Association between face memory and N170 latency								
1	Configural invariance	25.81	26	1	0.000	0.029		
2	Weak (metric) invariance	37.55	33	0.996	0.031	0.066	11.73 (7)	0.11
3	Strong (scale) invariance	38.63	38	0.999	0.011	0.066	1.07 (5)	0.96
Association between face memory and N170 amplitude								
1	Configural invariance	38.30	26	0.995	0.057	0.028		
2	Weak (metric) invariance	50.76	33	0.993	0.061	0.082	12.46 (7)	0.08
3	Strong (scale) invariance	52.45	38	0.994	0.051	0.082	1.69 (5)	0.89

Next, we report the regression weights between the latent variables of face memory and N170 latency for females and males, estimated simultaneously in the multiple group model with equal factor loadings in both groups (see Figure 5A). There was no significant association between face memory performance and N170 latency in women (β = −0.12, *P* = 0.22); in contrast, this relationship was stronger and reached statistical significance in men (β = −0.25, *P* < 0.01). To inferentially test whether the difference in brain–behavior relationships between the two groups was statistically significant, we ran a further multiple group model with equality constraints on loadings and intercepts, where we additionally fixed the regression weights between the latent factors face memory and N170 latency to equality across groups. The Δχ^2^ test with Δ*df* = 1 revealed that the observed difference in the regression weight between the two groups was statistically not significant (Δχ^2^ = 0.96, Δ*df* = 1, *P* = 0.32).

#### Relationship of face memory and N170 amplitude

The structural equation model testing the sex-independent contribution of the N170 amplitude to individual differences in face memory also showed very good fit to the data: χ^2^ [13] = 17.99, *P* = 0.16, CFI = 0.998, RMSEA = 0.036 and SRMR = 0.022. The brain–behavior relation revealed that—disregarding sex—individual differences in the N170 amplitude do not significantly contribute to individual differences in the accuracy of face memory (β = −0.07, *P* = 0.31).

In order to investigate sex differences in the relationship between face memory and N170 amplitude, we estimated the structural equation model for female and male groups simultaneously (see Figure 5B). Following the same rationale as for N170 latency, we tested measurement invariance of the structural models including the N170 amplitude. Results of all three models are shown in Table 1, bottom. All three models showed very good fit to the data and no significant differences between configural invariance and metric invariance models, as well as between metric invariance and scale invariance models. Therefore, measurement invariance holds across sexes also for N170 amplitude.

**Fig. 6:**
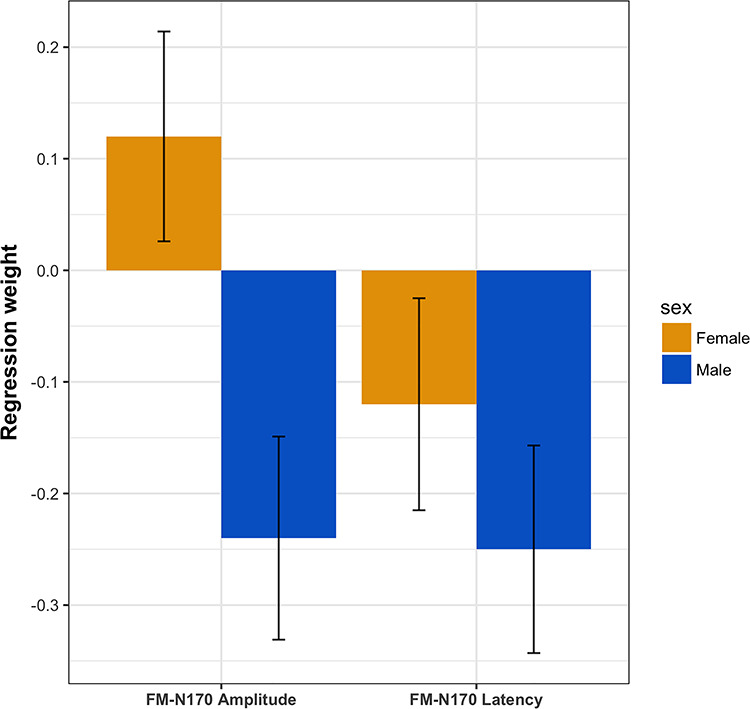
Summary of sex differences in brain–behavior relationships between face memory performance and N170 latency and N170 amplitude, shown as standardized regression weights.

The regression weights parameterizing brain–behavior relations separated for sex (see Figure 6) revealed no significant associations between face memory and N170 amplitude for females (β = 0.12, *P* = 0.20); in contrast, this relationship was present and significant for males (β = −0.24, *P* < 0.01). To inferentially test whether the difference between the two groups is statistically significant, in a multiple group model with equality constraints on loadings and intercepts, we additionally fixed the regression weights between the latent factors face memory and N170 amplitude to equality across groups. The Δχ^2^ test with Δ*df* = 1 revealed that the observed difference in the regression weights between the two groups was statistically significant (Δχ^2^ = 7.55, Δ*df* = 1, *P* < 0.01).

## Discussion

In the current study, we investigated whether women’s superior face memory relative to men can be traced to the speed and efficiency in structural encoding of faces—a mechanism reflected in the N170 component of the ERP. To this aim, with a large sample of 142 women and 152 men at hand, we investigated sex differences (i) in face memory accuracy at the behavioral level, captured with a psychometric test battery, (ii) in latency and amplitude of the N170 component at the neural level and (iii) in the association between accuracy of face memory and the N170 latency and amplitude.

### Confirmation of previous findings

Before discussing results about the neural mechanisms underlying sex differences in face memory, it is relevant to emphasize that the findings replicate previous reports about sex differences in face memory performance and N170 amplitude and latency. Thus, conforming with findings, for example, of Sommer *et al.* (2013), we observed a substantial superiority of women over men in face memory. We also found shorter N170 latencies in women as compared with men, replicating Choi *et al.* (2015) and Sun *et al.* (2017), and larger amplitudes, confirming Choi *et al.* (2015). Going clearly above these previous reports, the large sample and latent variable approach makes the present study well suited to estimate the true effect size of sex differences in face memory at behavioral and neural levels. The sex differences in N170 latency and amplitude are of medium effect size, putting the present findings on a solid basis. In line with current theorizing, these findings indicate that, on average, women show faster structural face encoding and engage more neural activity during face processing than men.

The overall relationships between face memory and N170 also conformed with expectations. As reported by Herzmann *et al.* (2010), there was a negative relationship between N170 latency and face memory abilities, that is, the shorter N170 latency the better the ability to memorize faces. Also in line with findings of Herzmann *et al.* (2010), there was no overall relationship of face memory with N170 amplitude, when females and males were not distinguished. Hence, individual differences in face memory abilities may at least partly be based on faster structural face encoding.

### Sex difference in the associations between face memory and the N170

The main question of the present study was whether the relationships between face memory abilities and the N170 parameters are sex-specific. To conduct such an analysis in a rigorous way, the invariance of the factorial structure across groups needs to be established at first. We therefore assessed configural, metric and scale invariance for both N170 latency and amplitude and found evidence for measurement invariance, that is, any sex difference cannot be due to a differential meaning of the measurements across groups. Therefore, not only the structure of face cognition abilities seems to be invariant for men and women (Sommer *et al.*, 2013) but also the factorial structure of the N170 parameters. Therefore, we are justified to assess the differential relationship between face memory and N170 parameters in women and men. By means of multiple group structural equation modeling, we split this relationship by estimating it separately but simultaneously, for females and males.

For N170 latency we found the negative relationship with face memory—seen across all participants when sex is disregarded—to be significant in men, whereas this association failed to reach significance in women. However, the seeming sex difference of this relationship failed significance. Therefore, the present results confirm the overall negative relationship between N170 latency and face memory—as mentioned—but do not allow to draw any conclusions about a sex difference in this particular brain–behavior relationship.

In contrast, N170 amplitude—which showed no significant general, sex-independent, relationship with face memory—showed a significant negative relationship with face memory, specifically in men. The numerically positive relationship in women was not significant as such but differed significantly from the negative relationship in men. Hence men with relatively good face memory are characterized by large N170 amplitudes, whereas men with poorer face memory tend to show smaller amplitudes of this component. In terms of the neural mechanisms indicated by the N170 component, we may therefore conclude that face memory ability of high-performing men is related—at least partially—to relatively more intensive structural face encoding than in low-performing men. This is in line with previous studies which show, for example, larger N170 in older adults (e.g. Wolff *et al.*, 2012), for inverted faces (e.g. Eimer, 2000; Wiese, 2012) and for other-race compared with own-race faces (e.g. Wiese, 2012). These findings indicate that elevated resources have to be invested for structural encoding of faces in difficult conditions or to compensate age-related deficits.

Although the observed relationship between ERPs and performance is correlational and not causal, the fact that the N170 precedes the performance indicators in time and is generated within the core system of face processing (e.g. Deffke *et al.*, 2007) the individual differences in N170 amplitude might be causal for the individual differences observed in face recognition performance. Why was there no significant relationship between N170 amplitude and face memory in women? One clue may be based on the findings of Sommer *et al.* (2013) that face memory declined across age in men, but not in women, and that the decline in men was negatively related to their amount of social activities. Hence, the non-significant relationship of face memory and N170 amplitude in women may indicate that due to their higher interest in social activities, they tend to more exhaustively exploit their potential of developing good face memory, which may go along with a similarly exhaustive development of the structural face processing system, reflected in N170 amplitude.

### Strengths, limitations and perspectives

The present study has a number of strengths. Firstly, we could show differential relationships of N170 amplitude and face recognition performance for men and women in independent sessions and trials. That is, we can rule out that our findings are due to dependencies of data as they would emerge when both performance and ERPs stem from the same trials. Secondly, the differential relationships are present in data that are independent and functionally distant. The N170 is interpreted as indicator of a basic perceptual mechanism—structural encoding of faces—whereas the performance data come from a memory (recognition) task. Hence, we could show a relationship between an early neural process and a performance indicator that requires both perceptual and mnemonic processes. Hence, the present study extends our knowledge about sex-specific relationship between face memory and the N170. However, several limitations should be mentioned as well.

First, at the performance level, any mean sex differences are straightforward, indicating female superiority in face memory. This is, however, different for sex differences in N170 amplitudes at the group level because they might be confounded with head and brain size, which are somewhat smaller in women (e.g. Rushton and Ankney, 2009). Hence, larger N170 amplitudes in women could be related to the smaller distance of the recording electrodes to the neural generators and/or to greater neural activity involved in structural encoding. Although the head size account for group differences in N170 amplitude cannot explain differential brain–behavior relationships across groups, future research should consider individual head size and electrical source modeling.

Second, by using the same face images at learning and recognition, what we describe as face memory in the present study is, at least in part, face image memory, which is presumably an overestimation of individuals’ true ability to recognize unfamiliar faces in real life (e.g. Young and Burton, 2017). However, there is no empirical evidence to date indicating that the rank order of individuals is different for same image *vs* different image face recognition tasks. The difficulty of different image recognition tasks is arguably larger, but it remains to be tested whether individuals who are mastering the one are less able to master the other.

Third, because the measurement of EEG phenotypes is very time-consuming, studies often have lower sample size than desirable. Our attempt to integrate data across studies aimed to compensate for low sample size. However, the present studies were not alone and specifically designed for investigating sex differences. Therefore, replication of the current findings in a new, independent dataset is worth pursuing.

Nevertheless, we emphasize that the aim of the present analysis was to further investigate previously reported associations between N170 and face perception and memory. In these previous studies, we have shown a correlation between N170 latency and face perception and memory (Herzmann *et al.*, 2010; Kaltwasser *et al.*, 2014) and a female advantage in face perception and memory (Sommer *et al.*, 2013). Therefore, it appeared plausible to investigate next whether the female advantage is related to processes, reflected in the N170 component. Future work should address sex-specific relationships between face memory and ERP components that are considered as electrophysiological markers of face memory, in particular, the N250 and N250r.

## Conclusions

In conclusion, we show here for the first time a differential relationship across men and women between a neurophysiological process seen to indicate structural analyses in perception and performance in face recognition on the level of individual differences. Such a differential relationship was not to be expected from the available data because, although women show better face memory and shorter N170 latencies than men as a group, for N170 amplitudes, the negative relationship in men and the (numerically) positive relationship in women seem to have partially cancelled each other to non-significance when sex was not taken into account. The present findings may encourage research efforts to further elucidate the neural basis of individual differences in male face memory, providing a perspective for individualized training programs.
